# Bedside Medication Management: Pharmacy Technicians Managing Patient Medication Supply to Improve Nursing Productivity and Patient Safety

**DOI:** 10.3390/pharmacy13060165

**Published:** 2025-11-07

**Authors:** Tom W. Simpson, Duncan S. Mckenzie, Rosina G. Guastella, Michael J. Ryan

**Affiliations:** 1 BPharm, FSHP, FANZCAP, Statewide Hospital Pharmacy, Tasmanian Health Service, Hobart, TAS 7000, Australia; 2 School of Pharmacy and Pharmacology, University of Tasmania, Hobart, TAS 7000, Australia; 3 Advanced Pharmacy Australia, Collingwood, VIC 3066, Australia; 4 Duncan S McKenzie, BPharm (Hons 1), FANZCP, MSHP, Pharmacy Projects, Office of the Deputy Secretary, Community, Mental Health and Wellbeing, Department of Health, Hobart, TAS 7000, Australia; duncan.mckenzie@ths.tas.gov.au; 5 Formerly Pharmacy Department, Royal Hobart Hospital, Hobart, TAS 7000, Australia; 6 Rosina G Guastella, BPharm, PharmConsult Pty Ltd., Hawthorn, VIC 3122, Australia; r.guastella@pharmconsult.com.au; 7 BPharm, FSHP, MBA, AFCHSM, PharmConsult Pty Ltd., Hawthorn, VIC 3122, Australia; m.ryan@pharmconsult.com.au

**Keywords:** medication, technicians, safety, productivity, nurse

## Abstract

Audits of medication charts conducted by Royal Hobart Hospital Pharmacy revealed that dose omission was the most common medication error experienced by patients. Investigation of these errors also found that nurses spend significant time organising medication for inpatients. To address the issues contributing to these problems, an alternative model of medication management was implemented and tested. This model of bedside medication management involves medication supply managed by ward pharmacy technicians who review charts daily for changes to medicines and obtain the medicines needed for each patient. Outcomes on two intervention wards showed that the model, combined with technician involvement in controlled medicines stock management, resulted in 29.78 h of nursing time released to patient care per 20-bed ward per week, for an investment of 22.28 h of ward pharmacy technician time; a 75% reduction in delayed doses; a 44% reduction in missed doses; and an average decrease of two hours in the turnaround time for supply of inpatient medication. Introducing bedside medication management and controlled medicines stock management activities can release 1.34 h of nursing time to patient care for every hour of ward pharmacy technician time (at a lower hourly salary cost), decrease dose delays and omissions, and improve patient safety.

## 1. Introduction

Medication administration omissions are a substantial problem for patients and one of the most common medication administration errors that occur in hospitals [1]. The three types of medication dose omissions most frequently observed in hospital studies worldwide are unsigned dose administration records, patient refusal to take the dose, and the medication not being available on the ward [2]. A UK study which included 5708 patients from 320 wards in 37 hospitals found that, excluding valid clinical reasons, 30% of patients experienced medication administration omissions, with approximately half of these patients having refused medicines. Patients prescribed more than 20 medications were approximately five times more likely to have had omissions than patients prescribed one to four medications [3].

Medication dose omissions contribute to adverse patient outcomes, being associated with increased length of stay [4,5] and compromised patient safety. According to a UK study which examined medication safety incidents in the NHS, 18% of medication incidents that cause death or serious harm are caused by dose omission [6].

Timeliness of medication administration is also significant, as delayed doses can adversely affect patient safety. Research in this area suggests that improving the timeliness of administration for certain medications has the potential to reduce patient morbidity and mortality, reduce lengths of stay and hospital costs and improve therapeutic outcomes [7]. Some ‘time-critical medicines’ (e.g., insulin, movement disorder medicines) must be given without delay in order to avoid serious harm [8]. The systems used for medicine distribution in hospitals is a contributing factor to dose omissions and delays. An Australian study reported that although the imprest (ward stock) system is a low-cost method for medicine distribution in hospitals, it is a high-risk system, leading to errors in 20% of medication administrations, the majority of which are dose omissions [9]. In addition, ward stock systems impose significant workload burden on nursing staff, who need to determine which medications are held ‘on imprest’ and which need to be ordered for individual patients, often at the time that dose administration is meant to occur. This needs to be considered in the context of nursing workforce shortages. The Australian Government’s Nursing Supply and Demand Study 2023–2035 found a likely shortfall of 70,707 FTE nurses by 2025 [10].

Initiatives to reduce the incidence of dose omissions and delays have had mixed results. A service improvement project conducted by Sheffield Teaching Hospitals NHS Foundation Trust which aimed to increase the availability of critical medicines through changes in the work processes of the ward and pharmacy professionals failed to achieve the objective of 95% availability [11]. Projects involving pharmacy assistant-supported medicines administration activities have shown more positive results and provide evidence for a potential solution [2,12,13]. Studies involving ward pharmacy technicians (WPTs) and individual patient storage found that this model reduced administration errors by 75% [14], reduced medication costs by 5%, and significantly reduced dose omissions [15].

Statewide Hospital Pharmacy (SHP) is the unit within the Tasmanian Health Service (THS) that provides all medication management and pharmacy functions. SHP uses the imprest system for inpatient medication supply in hospital wards, augmented by the dispensing of non-imprest items as needed.

Annual audits of medication charts conducted by SHP had revealed that medication omission was the most common medication error experienced by patients of the THS. SHP’s investigation of these errors also found that nurses spend significant time undertaking tasks associated with organising medication supply for inpatients at the expense of direct patient care activities.

This is particularly noteworthy in the context of significant workforce nursing shortages (both across Australia and within the State of Tasmania), and in this context, opportunities to streamline nursing tasks need to be considered. The role of pharmacy technicians in substituting or augmenting pharmacist roles is an established practice [16], but the impact on nursing ‘time to care’ has not been as widely studied.

The main aim of this study was to identify if an alternative model of medication distribution could reduce dose omissions and the time that nurses spend undertaking tasks associated with organising medication for inpatients at the expense of direct patient care activities.

## 2. Materials and Methods

To address the issues contributing to medication omission, delayed dosing, and time spent by nurses organising inpatient medication supply, SHP considered a number of options to improve the system of inpatient medication supply in its acute hospitals. The options included automation, unit dose dispensing, and the bedside medication management (BMM) model, which was already well-established in a small number of hospital wards of the THS. BMM was selected as the option that provided the greatest opportunity to achieve the desired outcomes more broadly.

BMM involves holding patient-specific medications as close to the patient as possible (e.g., in bedside lockers, or patient-specific locations in medication trolleys), with Pharmacy taking responsibility for ensuring that sufficient supplies are available for each patient. BMM involves ward pharmacy technicians (WPTs) working weekdays and being responsible for the following:

- Checking the paper-based medication charts daily (Monday to Friday) for all patients on the ward;
- Manually ordering medications requiring dispensing from Pharmacy;
- Placing inpatient medication in designated ‘near-bedside’ patient-specific locations (whether sourced from Pharmacy, ward imprest, or the patient’s own supply);
- Annotating charts with the storage location of medicines (e.g., ‘fridge’);
- Organising medication held in medication trolleys/lockers/rooms;
- Restocking trolleys and imprest storage as required;
- Managing urgent imprest orders outside scheduled replenishment;
- Anticipating requirements and ensuring sufficient supplies over weekends.

SHP had previously conducted a six-week pilot evaluation of BMM in one medical and one surgical ward at the Royal Hobart Hospital (RHH). The analysis of the outcomes of the pilot programme determined that the BMM model warranted further adoption across the THS, with results including a 25% reduction in turnaround time for inpatient medication supply from Pharmacy.

In light of these positive results, SHP proposed an expanded pilot of BMM, which was approved by the THS Executive and conducted from late 2018 to early 2019.

A specialist independent consulting company was engaged by the THS to work with the SHP on assessing the benefits of the expanded pilot of a BMM model utilising WPTs (‘the study’). THS employed a competitive tender approach for the selection of the consultants, using a Request for Quotation (RFQ) to conduct a productivity impact analysis.

Criteria for selection (as described in the RFQ) included the respondents’ knowledge of public hospital pharmacy services and medication management systems, contemporary hospital inpatient medication distribution systems, time and motion and similar methodologies, and hospital inpatient nursing practice, particularly as it relates to inpatient medication management. No conflicts of interest were declared, and the consultants who conducted the project were experienced hospital pharmacists.

As the BMM model was already well-established in the service, the work to evaluate its benefits was undertaken as a quality improvement (QI) project and local policies at the time did not require Ethics Committee approval for QI projects such as this. As such, the work was exempted by the Tasmanian Department of Health from Ethics Committee review.

An additional component to the study was an assessment of the potential nursing productivity gain, which could be achieved by extending WPT responsibilities to include providing assistance with controlled medicines, i.e., schedule 8 (S8) and schedule 4D (S4D) medicine management activities, both of which have special requirements regarding their supply, storage, and records of transactions.

Under the expanded pilot, the BMM model was implemented in the Assessment and Planning Unit (APU) and the Cardiology Unit (2D) of the RHH. Each unit has 20 beds, and involved the allocation of one WPT per ward, each working 0.5 FTE from 8.30 a.m. to 12.30 p.m. Monday to Friday (in total, equivalent to 1.0 FTE WPT working a 38 h week). The technicians selected as the WPTs for the pilot were experienced pharmacy technicians and the duties and responsibilities of the role fell within their existing scope of practice.

The objectives of the study were to evaluate the impacts of an expanded BMM model at the RHH on nursing productivity, patient safety, and quality use of medicines (QUM).

In line with the objectives, the primary outcome variable relating to nursing productivity was the nurse time involved in medication administration and related activities, while the secondary outcome variable relevant to patient safety and QUM was the availability of prescribed medication at the time a dose is required to be given. As such, the study methodology involved developing and testing an audit approach which included time and motion observations of time spent by nursing staff on dose administration to patients and activities related to medication supply.

The time and motion observations were conducted by three independent consultants using a structured data collection/observation tool, designed to facilitate easy recording of the pertinent information. The consultants were briefed on the use of the tool so that a consistent approach could be applied in recording the observations made. Each data collection sheet was supported by a scanned copy of the patient medication chart relating to the occasion of dose administration which was observed. The completed observation tools and accompanying charts were reviewed by a fourth consultant (also a pharmacist) who was not involved in the observations/site visit.

Two independent consultants made assessments regarding the following:

(a) Nursing productivity, assessed as follows:
  - Observing, measuring, and documenting activities and time involved in medication administration for a three-day period before BMM implementation, and again for a three-day period approximately five weeks after implementation. The three consultants rotated between wards and dose administration times to ensure that workload/observation time was shared equally across both wards, so that the impact of any variation in observer methods or potential inadvertent bias was reduced as much as possible.
    - A total of 160 occasions of patient dosing were observed, in which a total of 661 doses of medications were administered.
    - The time taken for the nurse to undertake all necessary steps of dose administration was recorded (e.g., including the time spent retrieving the medication from its location on the ward and checking it prior to administration).
    - Additionally, nursing time ‘lost’ to patient care during the dose administration process was recorded, which was defined as ‘unproductive’ time not essential to administering the charted medicine. This included any time a nurse was observed searching for medication potentially in various locations, ordering medicines (involving scanning the patient’s medication chart and emailing the scan to Pharmacy), or putting away medicines following delivery by Pharmacy.
  - Recording the medication requirements of patients in the pilot wards by making copies of each medication chart.
  - Measuring the time nurses spent managing S8 and S4D medicines requirements, including performing the end of shift ‘S8 count’ and obtaining ad hoc supplies of S8 and S4D medication, collecting these from Pharmacy, and completing an entry in the S8 or S4D register with a second nurse.
(b) Patient safety and QUM, assessed as follows:
  - Measuring the average turnaround time for the medication order delivery, defined as the time taken for a medication order to progress from the ward scanner to the dispensary, be dispensed by the Pharmacy team, and delivered to the ward.
  - Documenting delayed doses observed (i.e., the number of occasions that a dose of medication was not available at the scheduled administration time).
  - Documenting missed doses identified from a point prevalence audit of copies of medication chart dose administration records for each patient in the pilot wards during the time and motion visits.

The point prevalence audit involved reviewing the records to identify occasions where a scheduled dose administration fulfilled any of the following criteria:

- Remained unsigned or was marked as ‘N’ for ‘not available’. These doses were classified as ‘missed’.
- Signed, but the documented time of administration was significantly later than the prescribed dosing time. These doses were classified as ‘delayed’.
- Due at 8.00 a.m. (on the day of data collection) but remained unsigned at the time the chart copy was made (which was 10.00 a.m.). These doses were classified as ‘delayed’.

(c) Safety and security of medication storage areas, assessed by performing observations and conducting spot checks during the site visits.

## 3. Results

Table 1 and Table 2 present a summary of the individual elements comprising the key research objectives, with Table 1 outlining the nursing productivity impacts, and Table 2 outlining the benefits to safety (impact on delayed or omitted doses) and quality (timeliness of processes).

Table 3, Table 4, Table 5 and Table 6 present the data which informed the analysis. More detail on the results and related findings is provided in Section 3.6, Section 3.7, Section 3.8 and Section 3.9.

Table 7 combines all productivity-related measures to present the total magnitude of impact of the BMM system on a 20-bed ward, compared with the total pharmacy technician resource allocation to provide that service. Bolded figures in the body of Table 3, Table 4, Table 5, Table 6 and Table 7 highlight outcome measures used to calculate productivity benefits (see Table 1).

### 3.1. Results Analysis: Nursing Productivity, i.e., Nursing Time Released to Patient Care

In regard to nursing productivity, analysis results show that the BMM model had a positive impact in terms of releasing time to patient care, as a result of reductions in the following areas:

- Time spent on dose administration;
- Time ‘lost’ during dose administration;
- Time spent ordering medication from Pharmacy;
- Time spent on S8 medication checks.

Table 1 summarises the effect of implementing the BMM system on nursing productivity across the two pilot wards. Elements include nurse time spent on medication administration, scanning charts and ordering processes, end-of-shift checks, and ad hoc Schedule 8 (S8) medication collections. Columns show baseline values before BMM implementation (pre-BMM, A), values after BMM implementation (post-BMM, B), and the change (C = B − A). Negative values in the ‘Change’ column indicate time savings or reductions in workload The final column presents an estimate of the productivity impact across both wards, expressed as minutes gained per day.

**Table 1:** Individual elements impacting nursing productivity: quantitative analysis, measurement approach, and key results.

Element Description	Pre-BMM A	Post-BMM B	Change C (=B − A)	Impact (Across Both Pilot Wards)
(1) Nurse time spent on dose administration [minutes per medication given (avg)—see Table 3]	1.93	1.48	−0.45	239.85 min gained per day [=1C × 3B]
(2) Nursing time ‘lost’ ^1^ during dose administration [minutes per medication given (avg)–see Table 3]	0.33	0.05	−0.38	149.24 min gained per day [=2C × 3B]
(3) Number of scheduled doses in 24 h (see Table 4)	531	533		
(4) Nurse time spent scanning and sending scans of charts to Pharmacy to order medication [minutes per scan (avg)]	2.2	1.2	−1.0	51.03 min gained per day [= (4B × 5B/14) − (4A × 5A/14)]
(5) Number of scanned orders received by Pharmacy (from both pilot wards) over 14-day data collection period	520 (371 M-F, 149 S-S)	358 (250 M-F, 107 S-S)	−162 (−121 M-F, −40 S-S)	
(6) Nurse time spent on end of shift ‘S8 and S4D check’ [minutes per day (avg) (see Table 5 and Table 6)]	292.8	244	−48.8	48.8 min gained per week day ^2^
(7) Nurse time spent collecting ad hoc S8 orders from Pharmacy ^3^ (minutes per day, per ward)	25	nil		50 min gained per weekday
(8) WPT time required to collect S8 orders from Pharmacy ^4^	n/a	15 min		

^1^ Nursing time ‘lost’ to patient care during the dose administration process, defined as ‘unproductive’ time not essential to administering the charted medicine. ^2^ Achieved by having WTP replace second nurse for one check per day, Mon–Fri on each ward. ^3^ Measured by observation to derive average time (mins) per occasion (estimated to occur once per day, for each ward). ^4^ Not measured—estimated to be 15 min—included in resource allocation described in Table 7. WPT = Ward Pharmacy Technician; S8 = Schedule 8 (controlled) medication; M-F = Monday to Friday; S-S = Saturday to Sunday; Source: Own elaboration.

### 3.2. Analysis: Safety and Quality

In regard to patient safety and quality use of medicines, analysis results show that the BMM model resulted in a decrease of 115 min (54%) in turnaround time, a 75% reduction in delayed doses, and a 43% reduction in missed doses.

Table 2 presents a summary of the analysis of data on elements related to safety and quality, including medication turnaround time, delayed doses, and missed doses. Turnaround time encompasses five sequential steps from order receipt by Pharmacy to ward delivery. Reductions in delayed and missed doses are shown both as absolute counts and percentages of total doses due.

**Table 2:** Elements impacting safety and quality: quantitative analysis, measurement approach, and key results.

Element Description	Pre-BMM A	Post-BMM B	Change C (=B − A)	Impact (Across Both Pilot Wards)
Turnaround time [i.e., time taken for steps (i) to (v) for orders received over 14-day audit periods]	212 min	97 min	−115 min	Decrease of 115 min (54%) in turnaround time
i. order received by Pharmacy ^1^				
ii. order processed and dispensing label printed ^2^				
iii. labelled product checked by pharmacist and ready for collection ^3^				
iv. product collected from Pharmacy ^4^				
v. product delivered to ward ^5^				
Delayed doses ^6^				
Occasions when medication dose was not available at scheduled time (see Table 3)	59	16	−43	75% reduction in delayed doses
Total number of medications due to be given across all dosing occasions observed (see Table 3)	365	371	6	
Number of delayed doses as % of doses due	16%	4%	−12%	
Missed doses				
Number of doses not given ^7^	42	24	−18	43% reduction in missed doses
Number of regular doses scheduled in 24 h (see Table 4)	531	533	2	
Missed doses as a percentage of doses due in 24 h	7.9%	4.5%	−3.4%	

^1^ Measured using time stamp from Pharmacy server for receipt of scanned chart. ^2^ Measured using time data from Pharmacy dispensing system (iPharmacy^®^). ^3^ Time recorded in log by Pharmacy staff. ^4^ Time recorded in log by Pharmacy staff. ^5^ Not measured. Estimated to be 20 min from time of collection, based on data from the 2017 pilot study. ^6^ As observed and noted during time and motion studies. ^7^ Measured from point prevalence audit of medication charts (copies) for all patients in pilot wards at time of time and motion studies (i.e., across all times and days during the time of the study, not just the occasions when dosing was observed). Source: Own elaboration.

### 3.3. Data: Observations of Dose Administration Occasions

Data from the time and motion observations of dose administration is presented in Table 3. Observation identified (and logic suggests) that the time required for dose administration is directly proportional to the number of medicines that need to be given to the patient at the scheduled dosing time. As such, the average time spent on, and ‘lost’ during, occasions of dose administration was calculated and is presented ‘per medication given’.

Table 3 summarises data from the observations of dose administration activities made by the observers pre-BMM (A), post-BMM (B), and the resulting change (C = B − A). Measures include the number of dosing occasions observed and the number of medications administered. Time taken for dose administration and time ‘lost’ is shown per dosing occasion and per medication given. Data are presented for all observations for each of the two pilot wards (APU and 2D). Time metrics are reported in minutes, with ranges and averages specified. Negative values in the change column indicate a reduction or decrease in the metric, post-BMM.

**Table 3:** Dose administration: delayed doses, time taken, and time lost.

Measure		Pre-BMM A			Post-BMM B			Change C (=B − A)		
		All	APU	2D	All	APU	2D	All	APU	2D
Delayed doses										
Number of patient dosing occasions observed (DOO)	Total	80	53	27	67	24	43	−13	−29	+16
Number of medications due to be given across all dosing occasions observed	Total	365	241	124	371	163	208	+6	−78	+84
Number of medications given (all occasions observed)	Total	306	199	107	302	126	176	−4	−73	+69
Number of medications which were not available for administration at time of dosing (i.e., delayed))	Total	59	42	17	16	7	9	−43	−35	−8
Time taken										
Time taken (minutes) for patient dosing (all occasions observed)	Total	592	430	162	446	240	206	−146	−190	+44
Time taken (minutes) per patient dosing occasion observed:	Range	2–24	3–24	2–12	1–20	2–20	1–10			
	Average	7.4	8.1	6.0	6.65	10.0	4.78	−0.75	+1.9	−1.22
Time taken (minutes) per medication given	Average	**1.93**	2.16	1.51	**1.48**	1.90	1.17	**−0.45**	−0.26	−0.34
Time ‘lost’										
Number of dosing occasions where time ‘lost’ was observed	Total	48	32	16	13	6	7	−35	−26	−9
	% of DOO	60%	60%	59%	19%	25%	16%	−41%	−35%	−43%
Total time ‘lost’ (across all occasions observed) (minutes)	Total	101.8	68.5	33.3	15.8	4.5	11.3	−86	−64	−22
Time ‘lost’ per patient dosing occasion (as observed) (minutes)	Range	0.4–10	0.4–10	0.5–9.9	0.5–3.1	0.5–1	0.5–3.1			
Time ‘lost’ (minutes) per medication given	Average	**0.33**	0.34	0.31	**0.05**	0.04	0.06	**−0.28**	−0.36	−0.25

BMM = Bedside Medication Management; DOO = Dosing Occasion/s Observed; APU = Assessment and Planning Unit; 2D = Cardiology Unit; Time taken = time spent by nurses on dose administration activity; Time ‘lost’ = nursing time ‘lost’ to patient care during the dose administration process, defined as ‘unproductive’ time not essential to administering the charted medicine; Source: Own elaboration.

### 3.4. Data: Profile of Pilot Ward Patient Medication Dosing Requirements

Data on all the patients in the pilot wards during the time and motion visit was collected by observers making a scanned copy of each patient’s medication chart at a selected time during the course of each visit. The information was compiled to construct a profile of the medication needs of the ward’s patients at the time of the study, so as to provide context for, and support interpretation of, the study findings, and to facilitate the extrapolation of findings from the study sample to the broader patient group, i.e., all of the patients in the pilot wards at the time.

The information collected, which is presented in Table 4, shows a slight increase in patient numbers but a relatively consistent profile of patient dosing requirements in the pilot wards across the two audit periods.

Table 4 presents data on the number of medications (and doses) charted for administration, for all patients in the pilot wards during the time and motion visits conducted before and after the implementation of BMM, presented in the columns headed pre-BMM (A) and post-BMM (B), respectively. The difference between the two, calculated as (B) minus (A) is shown in the change column. Measures include the number of patients in the ward at the time of data capture, the number of medications charted for regular, scheduled administration (i.e., excluding ‘prn’ doses), the number of scheduled doses and dosing occasions in 24 h, and the number of medicines due to be given per patient dosing occasion. Measures of medications, doses and dosing occasions are reported as totals, with ranges and averages specified where applicable. Negative values in the Change column indicate a re-ductions or decrease in the metric, post-BMM.

**Table 4:** Profile of pilot ward patient medication dosing needs.

Measure		Pre-BMM A			Post-BMM B			Change C (=B − A)		
		All	APU	2D	All	APU	2D	All	APU	2D
Number of patients on ward at time of data capture	Total	36	24	12	41	27	14 ^1^	+5	+3	+2
Number of medications (regular) charted	Total	337	236	101	355	244	111	+18	+8	+10
	Range		2–19	3–19		1–24	1–18			
	Av. per pt	9.4	9.8	8.4	8.7	9	8	−0.7	−0.8	−0.4
Number of regular doses scheduled to be given over 24 h	Total	**531**	366	165	**533**	353	180	+2	−13	+15
	Range	3–30	3–30	3–28		1–28	1–38			
	Av. per pt	14.75	15.25	13.75	13	13	12.85	−1.75	−2.25	−0.9
Number of patient dosing occasions in 24 h	Total	156	100	56	166	113	53	+10	+13	−3
Number of patient dosing occasions (in 24 h) involving the administration of	1–2 meds	89	56	33	99	66	33	+10	+10	Nil
	3–5 meds	35	22	13	36	25	11	+1	+3	−2
	6–9 meds	19	11	8	20	15	5	+1	+4	−3
	≥10 meds	13	11	2	10	6	4	−3	−5	+2
Number of medicines due per patient dosing occasion	Range		1 to 15	1 to 13		1 to 16	1 to 17			
	Average	3.4	3.7	2.9	3.2	3.2	3.4	−0.2	−0.5	+0.5

^1^ There were 16 patients on the ward including two aged <18 yrs—data were not captured for these patients. BMM = Bedside Medication Management; APU = Assessment and Planning Unit; 2D = Cardiology Unit; All-data for both APU and 2D, combined; Medications (regular) = medications prescribed for regular, scheduled administration, e.g., four times a day; PRN = meaning ‘as needed’. A medication prescribed as PRN is not taken on a fixed schedule but only when necessary; Source: Own elaboration.

### 3.5. Data: Time Taken for S8 and S4D Counts

To inform the assessment of the potential nursing productivity gain that could be achieved by extending WPT responsibilities to include providing assistance with the management of controlled medicines (S8 and S4D), the study included time and motion observations of ‘end of shift’ drug counts. The process involves a physical check and count of the controlled medications stored in the ward safe and a reconciliation of the counted quantity with the balance recorded in the controlled drugs register. These counts are conducted by two nurses, on each ward, at the end of each shift (i.e., three times per day).

Table 5 presents data relating to the time spent by nurses on controlled drug counts, as measured by the observers. The table shows the number of counts observed, the ward location, and the time of day the observations were made. The time taken (in minutes) for the counts observed is presented as totals, with ranges and averages specified.

**Table 5:** Time taken by nurses conducting S8 and S4D counts at end of shifts.

Measure Shift Time		2D			APU			All Data		
		AM	PM	Night	AM	PM	Night	AM	PM	Night
Number of ‘end of shift S4D and S8 counts’ observed	Total	1	3	1	1	2	1	2	5	2
Time taken (minutes) to complete ‘end of shift S4D and S8 counts’	Total	32	61	27	26	45	29	58	106	56
	Range	32	16–27	27	26	20–25	29	26–32	16–29	27–29
	Avg	32	20.33	27	26	22.5	29	29	21.2	28
Average time taken (minutes) to complete an ‘end of shift S4D and S8 count’, across all shift times		25 min			24 min			**24.4 min**		

Table 6 applies the time and motion observation from Table 5 and applies it to the total number of S8 and S4D counts occurring across both pilot wards, noting that pre-BMM each count was conducted by two nurses, and post-BMM, the number of nurses required to undertake the count was reduced by one nurse per ward.

**Table 6:** Nursing time saved from WPT involvement in ward S8 and S4D count.

Measure	Pre-BMM	Post-BMM	Change
Number of S8 and S4D counts per day	6 (3 per ward)	6 (3 per ward)	
- Number performed by two nurses	6	4	
- Number performed by one nurse + one WPT	0	2	
Time taken for each nurse to perform one S8 and S4D count (from Table 4)	24.4 min	24.4 min	
Nursing time taken for S8 and S4D counts	(6 counts × 2 nurses) × 24.4 min = 292.8 min	([4 counts × 2 nurses] + [2 counts × 1 nurse]) × 24.4 min = 244 min	**48.8 min**

### 3.6. Summary of Results and Analysis: Nursing Productivity

Results analysis and data presented in Table 1, Table 2, Table 3, Table 4, Table 5 and Table 6 show that an increase in nursing productivity, i.e., nursing time released to patient care could be achieved by the BMM in the following ways:

- Reduction in time spent on dose administration:
  - The average time spent by nurses on dose administration was reduced from 1.93 min per medication in the pre-intervention period, to 1.48 min per medication in the post-intervention period (due mostly to nurses only having to handle current medication), i.e., a reduction of 0.45 min per medication (see Table 3).
  - Application of this time saving to the number of scheduled, regular doses in 24 h across the two pilot wards (see Table 4) yields a productivity gain of 239.85 min (3.99 h) per day over both wards (see Table 1) or 119.92 min (1.99 h) per day per 20-bed ward.
- Reduction in time ‘lost’:
  - The incidence of time being ‘lost’ during dose administration fell from 60% of dosing occasions observed in the pre-intervention period, to 19% of dosing occasions observed in the post-intervention period (see Table 3).
  - There was a corresponding reduction in the amount of time ‘lost’ of 0.28 min per medication given (from 0.33 to 0.05 min)—see Table 3.
  - Applying this to the total number of regular doses due in 24 h across the two pilot wards yields a time saving of 149.24 min (2.48 h) per day over both wards (see Table 1), or 74.62 min (1.24 h) per day, per 20-bed ward.
- Reduction in time spent scanning and sending charts to Pharmacy:
  - The introduction of the BMM model led to a reduction in the time spent by nurses sending scanned orders to Pharmacy of 63.2% (from 81.71 min to 30.63 min) on weekdays and 59.2% on weekends, despite the WPT working only on weekdays (see Table 1). This equates to a productivity gain of 51.03 min (0.85 h) per day across both wards, or 25.51 min (0.43 h) per day, per 20-bed ward.
- WPT assistance with S8/S4D management:
  - When a WPT replaced the second nurse in counting S8 and S4D medications during one shift on weekdays, the average amount of nurses’ time that was released to patient care was 24.4 min per weekday, per ward (see Table 1 and Table 6).
  - The average time spent by nurses collecting ad hoc orders for S8 and S4D medications from Pharmacy was 25 min per occasion (ranging from 21 min to 29 min). If this task was performed by a WPT once each weekday, the WPT could release approx. 25 min per weekday per 20-bed ward (see Table 1).
  - The combined productivity gain from having a WPT support these two activities was estimated to be 49.4 min (0.82 h) per weekday, per 20-bed ward, for an investment of 39.4 min (0.66 h) of WPT time per day.

Extrapolation of the productivity gains identified by the quantitative analysis, (see Table 7) shows that WPTs operating a BMM model and supporting S8/S4D management can release 29.78 h of nursing time to patient care, per week (7 days), per 20-bed ward. This release of nursing time can be achieved for an investment of 22.28 h of WPT time per week (Monday to Friday), which equates to 1.34 h of nursing time released to patient care for each hour of WPT time.

Table 7 presents a summary of the productivity gains identified by the quantitative analysis described in Table 1, extrapolated over a 7-day week for a 20-bed ward.

**Table 7:** Extrapolation and summary of time released to patient care.

Description of Productivity Gain	Estimated Time (h) Released to Patient Care, per Day (for a 20-Bed Ward)	Estimated Time (h) Released to Patient Care, per 7-Day Week (for a 20-Bed Ward)	WPT Time (h) Invested per 7-Day Week for a 20-Bed Ward
Reduction in nursing time: spent on dose administration	1.99	13.99	19.00 #
‘lost’ during dose administration	1.24	8.70	
spent scanning and sending scans of charts to Pharmacy	0.43	2.98	
spent conducting end-of-shift S8 checks (if WPT acts as second person for afternoon shift check, M-F)	0.41 *	2.03 *	2.03 *
spent collecting ad hoc S8 orders from Pharmacy (assuming WPT does this once per day each weekday)	0.42 *	2.08 *	1.25 *
**Total**	**4.49 (M-F)**	**29.78**	**22.28**
	**3.66 (S-S)**		

* Time released (and invested) Mon-Fri only. # 0.5 FTE working a 38 h week, Mon-Fri. M-F = Monday to Friday; S-S = Saturday and Sunday; S8 = Schedule 8 (S8) controlled drugs (e.g., morphine); Source: Own elaboration.

### 3.7. Summary of Results and Analysis: Patient Safety and QUM

Assessment of the impact of BMM on patient safety and QUM was based on the secondary outcome variable, which was the availability of the prescribed medication at the time a dose is required to be given. This was assessed through the measurement of turnaround time for patient medication orders, and the incidence of delayed and missed doses, before and after BMM implementation. Results of these measures are presented in Table 2).

- Reduction in delayed doses:

Data from the time and motion observations showed a reduction in medication doses which were not available for administration at the scheduled dosing time, dropping from 16% (59 of 365 medication doses due) pre-intervention to 4% (16 of 371) in the post-intervention period, i.e., a 75% reduction in delayed doses (as a proportion of the medication doses due to be administered).

- Reduction in missed doses:
  - The point prevalence audit of patient charts identified a decrease in the incidence of missed medication doses in the post-BMM period. Results showed that pre-intervention, 8% (42 of 531) of the doses scheduled to be given in a 24 h period were missed, compared with 4.5% (24 of 533) in the post-intervention period, equating to a decrease of 44%.
- Reduction in turnaround time:
  - The study showed a reduction of 115 min in average turnaround time under the BMM model (from 3 h 12 min pre-intervention to 1 h 17 min post-intervention), i.e., the required medications being available for administration to patients almost two hours earlier as a result of BMM.

### 3.8. Other Findings: Impact on Safety and Security of Medication Storage Areas and Wastage

Medication storage security: Improvements observed by the consultants in the post-intervention period included medication trolleys being locked more frequently (i.e., increased security); smaller volumes of stock being stored on shelves in cupboards facilitating easier (and faster) access for nurses to locate the medications they required; and no expired or inappropriately stored medications being found in the medication storage areas. These improvements in medication storage contribute to patient safety by reducing the opportunity for medication selection errors.

Wastage: It was observed that the WPT model allowed for greater predictive accuracy in the number of medications that a patient needed, reducing both overall waste as well as the time spent re-ordering medications that had been supplied in insufficient quantities.

### 3.9. Other Findings: Implications for Pharmacy

In this model, WPTs were employed by the pharmacy department specifically to provide benefits to staff and patients outside the pharmacy department. It is unlikely that the 22.28 h of WPT time per ward invested by the pharmacy is repaid in efficiency gains within the pharmacy department itself; although anecdotal reports suggest a reduction in phone calls and ad hoc orders, which may reduce associated pharmacy workload. Nevertheless, no evidence exists to suggest that the BMM model delivered any efficiencies for the pharmacy department.

## 4. Discussion

The combined productivity benefits of WPT involvement in BMM and S8/S4D stock management activities released to patient care were a total of 29.78 h of nursing time per 20-bed ward, per week, for an investment of 22.28 h of WPT time per 20-bed ward, per week. This translates to 1.34 h of nursing time released to patient care for each 1.0 h of WPT time.

Based on the findings from the BMM study and in light of the significant shortage of nurses, the Tasmanian Government approved the BMM model for implementation in all THS hospitals. The 35 technician positions which were created are expected to generate approx. 1500 h each week of productive nurse time (i.e., patient care time) across the State.

The key facilitators to achieving a successful outcome included identifying and clearly describing the problems (including those beyond Pharmacy, e.g., nurse shortages), designing and implementing a pilot study, using the results from the pilot to generate interest and support from key Hospital decision-makers for an expanded pilot, and driving a rigorous methodology and project planning through the involvement of external consultants.

The limitations of the study include the following:

(a) As the study was conducted in two medical wards, the results may not be transferable to other hospital inpatient units and specialty areas.

If the study was repeated, one change that would be considered would be including additional hospital inpatient units and specialty areas beyond medical wards.

(b) The estimate of time released to patient care by having a WPT responsible for the collection of ad hoc and S8/S4D orders was based on an assumption that a WPT could complete this activity in 15 min (compared with the average 25 min, observed to be required for a nurse).
(c) Qualitative and quantitative methods were used to draw inferences from the data.
(d) Funding for the study was provided by the THS as a quality improvement activity.

## 5. Conclusions

This study provides novel evidence of the productivity benefit of a medication management system that has been shown to improve patient safety by improving timeliness of treatment and reducing medication administration errors and can be leveraged to create cases for change in other hospitals.

## Data Availability

The original contributions presented in this study are included in the article. Further inquiries can be directed to the corresponding author.

## Abbreviations

The following abbreviations are used in this manuscript:

APU	Assessment and Planning Unit
BMM	Bedside medication management
DOO	Dosing occasions observed
FTE	Full Time Equivalent
QI	Quality improvement
QUM	Quality use of medicines
RHH	Royal Hobert Hospital
S8	Schedule 8 medicines are controlled drugs with a high potential for misuse, abuse, and dependence. These include opioid analgesics such as morphine and fentanyl
S4D	Schedule 4 Appendix D medicines as listed in the Poisons Act 1971 (Tas.) and the Poisons Regulations 2008 (Tas.) are controlled drugs which include drugs which may be abused and/or are liable to cause dependence
SHP	Statewide Hospital Pharmacy
THS	Tasmanian Health Service (THS)
WPT	Ward pharmacy technician
